# Deep dive into the immune response against murine mesothelioma permits design of novel anti-mesothelioma therapeutics

**DOI:** 10.3389/fimmu.2022.1026185

**Published:** 2023-01-04

**Authors:** Esther Stern, Stefano Caruso, Clément Meiller, Inbal Mishalian, Theo Z. Hirsch, Quentin Bayard, Carmit T. Tadmor, Hanna Wald, Didier Jean, Ori Wald

**Affiliations:** ^1^ Gene Therapy Institute, Hadassah Hebrew University Medical Center and Faculty of Medicine, Hebrew University of Jerusalem, Jerusalem, Israel; ^2^ Centre de Recherche des Cordeliers, Inserm, Sorbonne Université, Université Paris Cité, team Functional Genomics of Solid Tumors, Paris, France; ^3^ Tel Aviv University, Tel Aviv, Israel; ^4^ Department of Cardiothoracic Surgery, Hadassah Hebrew University Medical Center and Faculty of Medicine, Hebrew University of Jerusalem, Jerusalem, Israel

**Keywords:** immunotherapy, lung neoplasms, thoracic cancer, animal models, immunomodulation

## Abstract

Given the need to improve the efficacy of standard-of-care immunotherapy (anti-CTLA-4 + anti-PD-1) in human malignant pleural mesothelioma (hMPM), we thoroughly characterized the immunobiology of the AB12 murine mesothelioma (MM) model, aiming to increase its accuracy in predicting the response of hMPM to immunotherapy and in designing novel anti-hMPM treatments. Specifically, we used immunologic, transcriptomic and survival analyses, to synchronize the MM tumor growth phases and immune evolution with the histo-molecular and immunological characteristics of hMPM while also determining the anti-MM efficacy of standard-of-care anti-hMPM immunotherapy as a benchmark that novel therapeutics should meet. We report that early-, intermediate- and advanced- AB12 tumors are characterized by a bell-shaped anti-tumor response that peaks in intermediate tumors and decays in advanced tumors. We further show that intermediate- and advanced- tumors match with immune active (“hot”) and immune inactive (“cold”) hMPM respectively, and that they respond to immunotherapy in a manner that corresponds well with its performance in real-life settings. Finally, we show that in advanced tumors, addition of cisplatin to anti CTLA-4 + anti PD-1 can extend mice survival and invigorate the decaying anti-tumor response. Therefore, we highlight this triple combination as a worthy candidate to improve clinical outcomes in hMPM.

## Introduction

Human malignant pleural mesothelioma (hMPM) is a highly aggressive cancer for which immunotherapy with nivolumab + ipilimumab (anti PD-1 + anti CTLA-4, respectively) is the standard-of-care. Unfortunately, therapy response rates do not peak above 40% and median survival periods do not exceed 18 months (1, 2). Therefore, combinations of anti-hMPM immunotherapy with or without other treatments that will show benefit over and above the standard-of-care are highly desired (3).

To test and design new anti-hMPM therapies, asbestos-induced syngeneic murine mesothelioma (MM) cell lines were developed (4–7). However, only minority of treatment protocols designed using these preclinical cell line based MM tumor models showed sufficient clinical benefit to be broadly adopted (5). A key question is how to better utilize such MM tumor models to properly select effective therapeutics.

MM tumor models are appealing for immunotherapy research because they mimic the histological spectrum of hMPM and because they provoke an anti-tumor response that is responsive to immunotherapy within a week from implantation (8). Illustrating their utility, previous research using these models has highlighted impressive anti-MM effects of various combinations of checkpoint therapies together with chemotherapy or radiotherapy or surgery (9). For example, Fear et al. showed that combination of anti CTLA-4 together with anti OX-40 was synergistic in enhancing complete MM tumor regression (6). Similarly, Wu et al. and De La maza et al. respectively showed that administration of anti CTLA-4 between intervals of chemotherapy (8) or after radiotherapy and surgery (10) offered effective multimodal anti-MM therapeutics, successfully boosting the anti-tumor response.

And yet, the majority of studies in MM tumor models suffer from common limitations that preclude linear deduction of their findings to the clinic. First, in many studies, therapeutic interventions were tested relatively early following tumor implantation when the tumor has not yet imprinted its immunosuppressive effects in the tumor microenvironment (TME) nor remotely in the host (11, 12). This may yield an underestimation of loss of treatment efficacy in advanced disease phases which is almost always the state of hMPM at the time of diagnosis (13, 14). Second, so far, studies have not longitudinally compared the TME of MM tumors relative to that of hMPM, nor tested treatment efficacy across tumor growth phases that represent distinct immune activation states. Thus, optimal anti-MM effects were possibly detected under conditions that do not properly mimic hMPM. Third, studies did not routinely compare the efficacy of new immunotherapeutic combinations relative to that of the standard-of-care, which could result in selection of suboptimal candidates for translation to the clinic (6).

To address these issues, we herein report on a systematic investigational approach that we applied in the AB12 MM tumor model in order to improve its preclinical predictive capacity. First, specific AB12 tumor growth phases were determined to assure that what is tested are well-established tumors. Second, the immune response in the TME and at remote immune sites was characterized in each of the tumor growth phases to identify specific phases that are predominated by immune activation, immune transition and immune suppression. Third, the efficacy of standard-of-care anti-hMPM immunotherapy was determined in all tumor growth phases to serve as a preclinical benchmark for evaluation of new interventions. In parallel, in steps one and two, the histo-molecular and immunological characteristics of AB12 tumors were respectively compared to hMPM in order to permit synchronization of experimental conditions in the model with its counterpart human disease.

Of the wide variety of MM cell lines that are available for research (7, 15, 16) we selected to implement our investigational approach using the biphasic murine AB12 cell line for three reasons: (i) biphasic MPM is the most common histo-molecular subtype of hMPM, representing 50% of all human disease (17); (ii) previous studies have shown that AB12 cells are highly immunogenic in the manner in which they elicit an anti-tumor immune response (8, 10); and (iii) this study is focused on immunotherapy which nowadays is becoming the standard-of-care treatment for non-epithelioid hMPM (1, 18). We further selected to use young female mice in our experiments, despite the fact that hMPM is mainly diagnosed in elderly males and despite that fact the efficacy of immunotherapy is partly age dependent (19) because past research demonstrates striking histological similarities between biphasic hMPM and AB12 tumors that were transplanted in young female mice (4). This coupled with the fact that the vast majority of studies that examine the immune response associated with MM tumors have been performed in young female mice (5, 6, 8, 16, 20–24) will allow other researchers to more easily align past and future research with our findings.

Overall, in light of the surge of novel immunotherapeutic drugs that are anticipated to be approved in the near future (25), our study delineates a more standardized and systematic approach aiming to guide the utilization of MM models in preclinical studies.

## Methods

### Study design

This study was designed, using immunologic, transcriptomic and survival analyses, to explore a three-step approach that permits synchronization of tumor growth phases and of immune response in the MM model (both in tumor and in remote immune sites) with that of histo-molecular and immunological characteristics of hMPM while also determining in the MM model the efficacy of current standard-of-care anti-hMPM immunotherapy as a benchmark that novel therapeutics should meet.

### Mice

BALB/c mice were purchased from Envigo. 8-week-old female mice were used for all studies.

### Cell lines

The AB12 cell line was kindly provided by prof. Zvi Friedlander’s lab at Hadassah. AB12 cells were grown in DMEM culture medium (Gibco) supplemented with 10% fetal bovine serum (FBS) (Biological industries), 2 mM l-glutamine, penicillin (100 U/ml), streptomycin (100 ug/ml), neomycin (100 ug/ml) and sodium pyruvate (2mm) (BI). Cells were grown in a 37°C and 5% CO2 environment and were harvested when 70% confluent.

### Preparation of cell suspensions for flow-cytometry analysis

Peripheral blood (PB) was collected in tubes containing heparin. Spleens were harvested and passed through a 70-mm cell strainer to generate single cell suspension. Bone marrow (BM) cells were extracted from two femur bones by flushing with 1ml of cold PBS, using a 25G needle. Next, the PB, spleen and BM cell suspensions were treated with an erythrocyte lysis solution (155 mM NH_4_Cl, 10 mM KHCO_3_, 0.1 mM Na_2_EDTA, pH: 7.4). The cells were then washed and stained as described below.

### Isolation of tumor infiltrating cells

Fresh tissue was cut into 1 mm^3^ pieces and digested with 1.5mg/mL collagenase type IV (Worthington Biochemical) and 0.05 mg/mL DNase I (Sigma-Aldrich) at 37°C for 20 minutes. The dissociated tissues were filtered through a 70µm cell strainer and washed with DMEM culture medium (Gibco) supplemented with 10% FBS and centrifuged.

### Flow-cytometry

Staining was performed for 30 minutes at 4°C in PBS. All antibodies were purchased from Biolegend and were used in 1:50-1:100 dilutions. The complete list of antibodies is shown in **Supplementary Table 1**. The compensation control was performed using single color staining. Unstained controls and Fluorescence minus one (FMO) control were utilized to establish baseline gate settings for each respective antibody-fluorophore combination used in individual experiments. Stained cells were analyzed with CytoFLEX Cytometer (Beckman Coulter). Data analysis was performed using the Cytexpert software (Beckman Coulter).

### Tumor experiments

AB12 cells (1 × 10^5^) were injected to the peritoneum of BALB/c mice (at least 7 mice per group). To determine survival, mice were routinely monitored and when they developed significant ascites or their general condition deteriorated, they were euthanized. Tumor tissue was collected from the peritoneal cavity immediately after the mice were anesthetized. The percentage of necrotic area out of entire tumor area was determined in hematoxylin and eosin stained tumor section based on morphology using the ImageJ software.

#### CTLA-4, PD-1, LAG-3 and TIM-3 blockade and cisplatin treatment

200µg of anti CTLA-4 (clone 9D9), anti PD-1 (clone RPMI-14) anti LAG-3 (clone C9B7W) or anti TIM-3 (RMT3-23) blocking antibodies (BioXcell) were injected to the peritoneum of tumor-bearing mice at the indicated time points. The treatment protocol for all antibodies, unless otherwise stated in text, consisted of four injections: two injections per week for two weeks. 5mg/kg of cisplatin (Pharmachemie B.V.) was injected intravenously to tumor-bearing mice at the indicated time points. The treatment protocol consisted of two injections: one injection per week for two weeks.

### RNA isolation

Total RNA from tumor and spleen tissues was isolated using TRIzol reagent (Ambion) according to the manufacturer’s protocol, followed by Direct-zol (Zimo). The concentration and the quality were evaluated using a NanoDrop 2000c (Thermo scientific) and the RNA integrity number (RIN) was determined using Agilent Bioanalyzer (TapeStation RNA 2200) for RNA sequencing.

### Quantitative real-time RT-PCR

cDNA was synthesized from 1-2μg total RNA using Biosciences qScript cDNA Synthesis Kit (Quanta). Quantitative PCR (qPCR) was performed using the CFX384, C1000 touch thermal cycler (Bio-Rad), and a SYBR Green PCR Kit (Quanta). The fold expression and statistical significance were calculated using the 2^−ΔΔ^ *^Ct^* method. All experiments were performed in triplicate. Primers were purchased from IDT-syntezza. The primer sequences are listed in **Supplementary Table 2**.

### Co-culture and ELISA assay

For co-culture assays, mice were injected with AB12 cells, at the indicated time points prior to the experiment. Spleens from these mice were collected and splenocytes were isolated as described above. The number of viable splenocytes in each sample was determined by trypan blue staining. 24 hours prior to the experiment, 3 × 10^4^ AB12 cells per well were plated in flat-bottom, 24-well plates (Corning). Next, the medium was changed to fresh medium and splenocytes were added in 1:20 ratio. For cisplatin pretreatment, AB12 cells were plated 48 hours prior to the experiment and 24 hours later, the medium was changed to fresh medium either supplemented or not with 5μg/mL cisplatin. On the experiment day, the medium was replaced again by fresh non-cisplatin containing medium and splenocytes were added in 1:20 ratio. In both experiments, 24 hours following the co-culturing of tumor cell and splenocytes, the medium was collected for ELISA assay. IFN-gamma (IFNg) levels in culture medium were quantified using ELISA (DuoSet Mouse IFN-gamma immunoassay, R&D Systems) according to the manufacturer’s instructions.

### RNA sequencing

RNA-Seq was performed on AB12 tumors from at least 3 independent mice per group at the indicated time points. Libraries were prepared using the KAPA RNA HyperPrep kit (Roche), according to the manufacturer instructions. Paired-end 75 or 100 base massively parallel sequencing was then carried out on an Illumina NextSeq500 or NovaSeq6000, respectively. FASTQ files were aligned to the mouse reference genome GRCm38/mm10 using TopHat2 (v2.0.14). Uniquely mapped reads were kept and BAM files were indexed and sorted using Sambamba (v0.6.5). We used HTSeq to obtain the number of reads associated with each gene in the Gencode vM21 transcriptome indexes. The Bioconductor DESeq2 package was used to import raw HTSeq counts for each sample into R statistical software and to apply variance stabilizing transformation to the raw count matrix to obtain an expression matrix without variance-mean dependence (DESeq2-normalized counts). FPKM scores (number of fragments per kilobase of exon per millions of mapped reads) were calculated by normalizing the count matrix for the library size and the coding length of each gene. We removed 21,883 unexpressed genes (i.e., detected in less than 5% of samples) and an additional 4,102 genes with a significant batch effect (area under the ROC curve > 0.95 between one sequencing project and others).

### Human-mouse transcriptomic integrative analysis

A total of 306 samples from 3 different RNA-sequencing datasets were used for the comparative transcriptomic analysis, including 295 hMPM (from 2 different datasets: 209 and 86 hMPM from the Bueno and TCGA series, respectively) (26, 27) and 11 mouse AB12 samples. First, common genes that are 1:1 orthologs in human and mouse were selected using the list of mouse-human 1:1 orthologous genes from MGI (http://www.informatics.jax.org). Then, we filtered out most of the genes by keeping only the 2000 mouse-human 1:1 orthologous genes with the highest variance in human datasets. Finally, we standardized gene expression in each dataset to have mean 0 and standard deviation 1 per gene just before the three datasets were integrated. Unsupervised hierarchical clustering of the integrated data was performed using cosine distance and Ward’s linkage method on the 2000 most variant orthologous genes with ComplexHeatmap package in R statistical software. Histologic and molecular subtypes of hMPM were retrieved from Bueno et al. and Hjmeljak et al. (26, 27) and the histo-molecular scores were retrieved from Blum et al. (17).

### Pathway enrichment analysis

Differentially expressed protein coding genes between groups were determined using the DESeq2 package in R. Only genes with an adjusted *p*-value below 0.05 and a fold-change higher than 2 were considered. The hypergeometric test was used on overexpressed and underexpressed genes separately to identify enriched mouse Molecular Signatures Database gene sets (MSigDB v7.2.1 obtained using msigdb in R) in the list of differentially expressed genes. Over-representation analyses were performed using the Hallmark, KEGG, Reactome and GeneOntology databases. The over-represented signal pathways over-represented in several of the databases were grouped into families and sub-families, which are highlighted in **Supplementary Table 3**.

### Molecular classification and histo-molecular score predictions

Expression data from RNA sequencing (FPKM scores) were used to predict Thorsson subgroups (28). In particular, an ensemble classifier based on XGBoost was implemented to classify tumor samples into one of six immune subtypes using the “ImmuneSubtypeClassifier’’ package in R. Histo-molecular scores in human tumor samples were previously predicted (17). Only samples with a cumulated E.score and S.score higher than 50% were taken into account to ensure sufficient tumor content for correct estimation.

### Tumor microenvironment cell content

The human microenvironment cell population counter (MCP-counter) or mouse dedicated (mMCP-counter) methods were used to compute scores of infiltration for different immune and stromal cell populations from DESeq2-normalized RNA-seq data. MCP-counter scores were calculated using the MCP-counter method (29) previously validated in hMPM tumor samples (17) and adapted to RNAseq data using genes filtered on hMPM cell lines (30) whereas mMCP-counter scores were obtained using the mMCPcounter package in R (31). The list of specific genes used for each population is available in **Supplementary Table 4**. Infiltrations were determined using the scores of MCP-counter for hMPM and mMCP-counter for AB12 tumors and matching of the cell populations between the two predictive tools. A total of 295 human tumor samples from two different series of RNAseq were combined in the integrated analysis with public datasets, including the 209 samples from Bueno et al. (26) and 86 samples from TCGA (27). Then, we standardized gene expression separately to have a mean of 0 and a standard deviation of 1 per gene in each dataset. Statistical analysis and data visualization were performed using R software. Unsupervised hierarchical clustering was performed using cosine distance and Ward’s linkage method.

### Statistics

Statistical analysis was done using the GraphPad Prism software or R statistical software. Statistical significance was calculated using unpaired Student’s t-test for pairwise comparisons. For multiple comparisons, a one-way ANOVA test was performed, and pairwise significance was determined by Tukey’s multiple comparisons test. Statistical differences between survival curves were calculated by log-rank test. Values of *p* < 0.05 were considered statistically significant.

### Study approval

The Animal Care and Use Committee of the Hebrew University approved all experiments.

## Results

### PART 1. Determining the AB12 tumor growth phases and comparison to hMPM.

#### Determining the AB12 tumor growth phases

**Figure 1:**
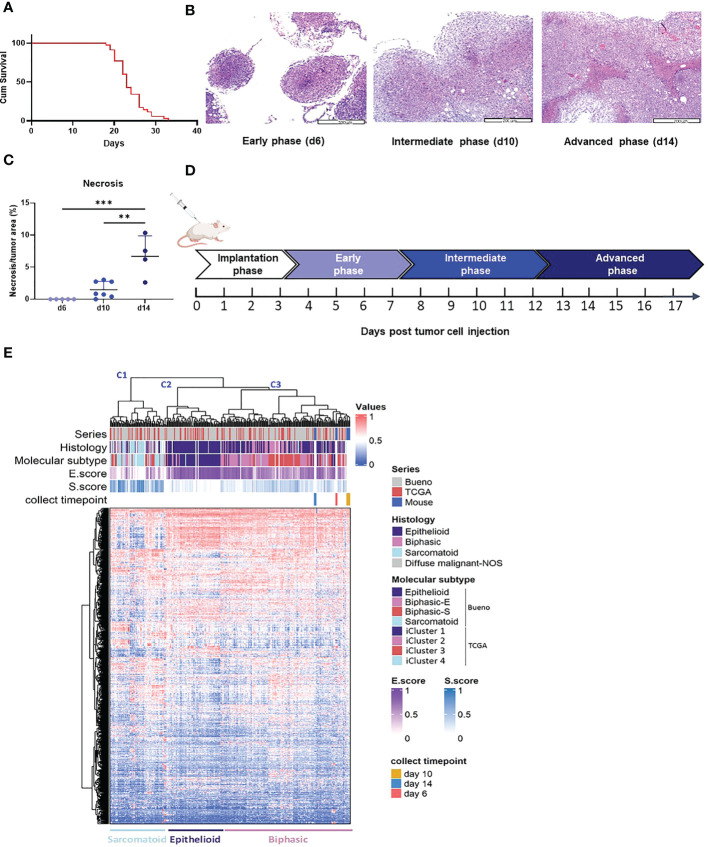
AB12 tumors characterization. **(A)** Kaplan-Meier survival curve of mice injected with AB12 cells. n=28. Cum: cumulative. **(B)** Histological features of early, intermediate and advanced phase AB12 tumors. Representative hematoxylin and eosin staining of tumors. Original magnification X10. **(C)** Evaluation of the necrosis area in AB12 tumors. The percentage of necrotic area out of the entire tumor area in d6, d10 and d14 tumors is shown. Values of the *post hoc* Tukey test are indicated at the top of the dot plots. **: *p* ≤ 0.01; ***: *p* ≤ 0.001. **(D)** Schematic axis of tumor development. **(E)** Integration based on transcriptomic data of AB12 tumors in hMPM tumors. Unsupervised clustering of d6, d10 and d14 AB12 tumors with 295 hMPM tumor samples was performed based on transcriptomic data obtained by RNA-Seq. The series of each tumor sample, the histologic and molecular subtypes, the histo-molecular gradients (E.score and S.score) and the collect timepoint of AB12 tumors are indicated by a color code or a color gradient at the top of the heatmap. Clusters C1 to C3 are indicated at the top as well as the deduced histologic subtypes at the bottom.

To define the AB12 tumor growth phases, we measured the survival of tumor bearing mice and we longitudinally sampled the tumor appearance across time (days 3, 6, 10 and 14). As shown in **Figure 1A**, mice survived between 20 to 30 days following tumor implantation. We found that day 6 (d6) was the earliest time point at which peritoneal tumor nodules were visible to the naked eye and collectable. As shown in **Figures 1B, C**, the histological appearance of the tumors was notable for small nodular implants on d6, for large and viable tumors on day 10 (d10), and for even larger tumors with multiple necrotic foci on day 14 (d14) (tumor diameter range: 2 to 4, 5 to 10, and > 12 mm, respectively). Based on these observations and given that the architectural patterns of the tumor developed in a gradual fashion, we defined the tumor growth phases as follows: days 0 to 3 (when no tumor implants are visible): “implantation phase”; days 4 to 7: “early-phase”; days 8 to 12: “intermediate-phase”; and days 13 and on: “advanced phase” (**Figure 1D**).

#### Comparison of AB12 tumors to hMPM

To compare the transcriptomic profile of AB12 tumors to that of hMPM, we performed unsupervised clustering of d6 (n=3), d10 (n=5) and d14 (n=3) AB12 tumors together with a cohort of 295 hMPM samples (**Supplementary Table 5**). As shown in **Figure 1E**, the series split into three main transcriptomic clusters, termed C1 to C3. Further, AB12 tumors— in all of their growth phases— belonged to cluster C3. Comparison of the distribution of the histologic and molecular subtypes in human tumor samples (26, 27) as well as the E.score and the S.score of the histo-molecular gradients (17) (**Figure 1E**, **Supplementary Figure 1A**) showed that tumors in clusters C1, C2 and C3 are sarcomatoid, epithelioid and biphasic, respectively, representing 23%, 24% and 52% of hMPM samples in the cohort.

### PART 2. Characterizing the tumor phase dependent immune response that AB12 cells provoke and alignment with immune states in hMPM

#### Characterizing the immune response that AB12 cells provoke in the TME

**Figure 2:**
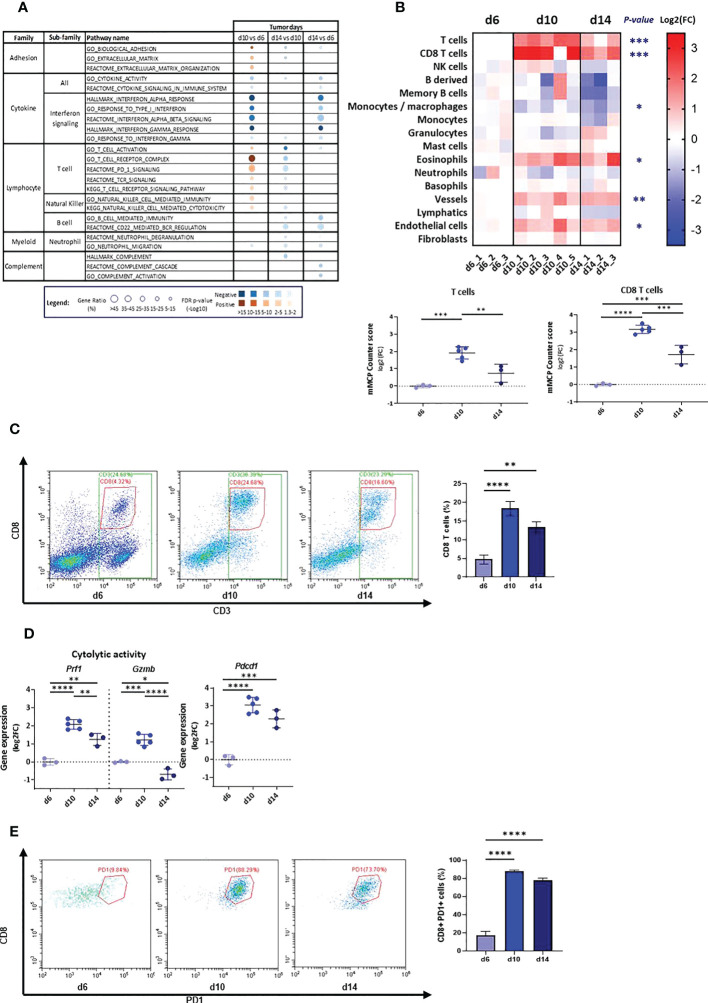
Tumor microenvironment in d6, d10 and d14 AB12 tumors. **(A)** Dysregulated signal pathways between tumors identified by over-representation analysis. The families and sub-families of the major over-represented signal pathways are shown in the figure for each comparison indicated at the top of the figure. Over-representation of each pathway, based on underexpressed and overexpressed genes, in blue and brown, respectively, is indicated as a circle, whose size is proportional to the gene ratio and the color gradient represents the FDR *p*-values. **(B)** Differential infiltration of immune and stromal cell populations between tumors. In the upper part, the fold-changes (FC) in mMCP-counter scores of each cell population are represented as a heatmap. The collect timepoints and the names of the AB12 tumor samples are indicated above and below the heatmap, respectively. The significant *p*-values of the ANOVA test are indicated at the left of the heatmap if the FC is higher than 2 in d10 or d14 compared to d6. In the lower part, the dot plots show the FC of T cell and CD8 T cell populations. The FC are relative to the mean scores obtained in d6 tumors. **(C)** T cell infiltration of AB12 tumors identified by flow cytometry. Single cell tumor suspensions, stained with anti CD45, anti CD3 and anti CD8 antibodies, were analyzed using flow cytometry. Representative FACS dot plots show the percentage of CD3+, CD8+ and CD3+ CD8-, corresponding to CD3+ CD4+ cells, out of CD45+ cells in the tumors. On the right, the bars show the average percentage of CD3+ PD8+ cells out of CD45+ cells in the tumors (n=3 per time point). **(D)** Differential expression of T cells cytolytic and activation genes between d6, d10 and d14 AB12 tumors. The dot plots show the FC relative to the mean of the d6 tumor gene expressions of *Prf1* (perforin-1), *Gzmb* (granzyme B) and *Pdcd1* (PD-1) genes, based on RNA-Seq data. **(E)** PD-1 expression on tumor infiltrating CD8 T cells identified by flow cytometry. Single cell tumor suspensions, stained with anti CD45, anti CD3, anti CD8 and anti PD-1 antibodies, were analyzed by flow cytometry. Representative FACS dot plots show the percentage of CD8+ PD-1+ cells out of CD3+ cells in the tumors. On the right, the bars show the average percentage of CD8+ PD-1+ cells out of CD3+ cells in the tumors (n=3 per time point). The *p*-values of the *post hoc* Tukey test are indicated at the top of the dot plots and of the histogram (B, D and E). FC: Fold-Change. *p ≤ 0.05; **p ≤ 0.01; ***p ≤ 0.001; ****p ≤ 0.0001.

To probe the immune response in the TME of AB12 tumors, we used pathway enrichment analysis and the mMCP-counter immune and stromal cell populations predictive tool (29, 30). We found that dysregulated pathways between the tumor growth phases were mainly related to the immune system (**Supplementary Table 3**). In particular, early-phase tumors were notable for relative enrichment in pathways associated with type I and type II interferon (IFN) responses whereas intermediate-phase tumors were notable for enrichment in pathways associated with T cell activation and signaling as well as pathways associated with natural killer (NK) immunity and cytotoxicity (**Figure 2A**, **Supplementary Figure 1B**). In line with the latter findings, the mMCP-counter tool showed that intermediate-phase tumors, relative to both early- and advanced-phase tumors, were enriched with T cells and CD8 T cells infiltration, with a higher fold-change for CD8 T cells between d6 and d10 (**Figure 2B**, **Supplementary Figure 1C**, **Supplementary Table 4**). Further, flow cytometry confirmed these findings and indicated that the increased infiltration of T cells is due to CD8 T cells (**Figure 2C**, **Supplementary Figure 1D**). Notably, gene expression analysis and flow cytometry demonstrated that the cytolytic and activation genes of T cells – perforin-1 (*Prf1*), granzyme B (*Gzmb*) and PD-1 (*Pdcd1*) – all peaked in expression in the TME of intermediate-phase tumors (**Figures 2D, E**).

#### Characterizing the immune response that AB12 cells provoke at remote immune sites

**Figure 3:**
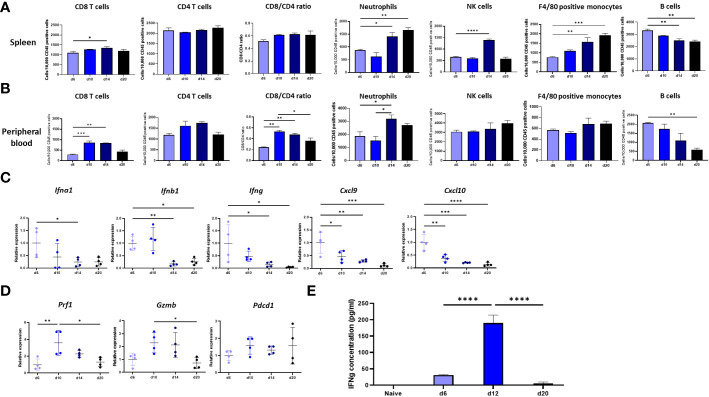
Immune response at remote immune sites. **(A, B)**. Changes in the immune content of the spleen and peripheral blood (PB) of tumor bearing mice between d6, d10, d14 and d20. The numbers, determined by flow cytometry, of CD8 and CD4 T cells, NK cells, neutrophils, F4/80 positive monocytes and B cells out of 10,000 CD45+ cells in the spleen **(A)** or peripheral blood **(B)** of tumor bearing mice are shown (n≥6). The CD8/CD4 ratio at both sites is shown as well **(C)** Tumor phase dependent expression in the spleen of genes that mark the anti-tumor response in the TME. The changes relative to d6 tumor mean expression of the *IFNa*, *IFNb1*, *IFNg*, *Cxcl9* and *Cxcl10* genes in the spleen of tumor bearing mice between d6, d10, d14 and d20 are shown (n=4). **(D)** Tumor phase dependent expression of cytolytic and activation genes in the spleen. The changes relative to d6 tumor mean expression of the *Prf1*, *Gzmb* and *Pdcd1* genes in the spleen of tumor bearing mice between d6, d10, d14 and d20 are shown (n=4). **(E)** Responsiveness of splenocytes derived from tumor bearing mice to AB12 cells *in vitro*. AB12 cells were co-cultured with splenocytes derived either from d6, d12 and d20 tumor bearing mice or from naïve mice and 24 hours later, the levels of IFNg protein in the medium were measured by ELISA (n=3). The *p*-values of the *post hoc* Tukey test are indicated at the top of the dot plots and the histogram (A to E). *p ≤ 0.05; **p ≤ 0.01; ***p ≤ 0.001; ****p ≤ 0.0001.

To characterize the immune response that AB12 tumors provoke at remote immune sites, we first determined the changes in immune content of the spleen, peripheral blood (PB) and bone marrow (BM) between our baseline at d6 and that at d10, d14 and d20. **Supplementary Figure 2A** shows the gating strategy to evaluate the presence of each immune cell population. We found that in the spleen, the number of CD8 T cells increased between d6 to d14 (*p* < 0.05), whereas that of CD4 T cells was relatively stable. Consequently, the CD8/CD4 ratio in the spleen tended to tilt more towards CD8 on d10, d14 and d20 than on d6 (**Figure 3A**). We also found that the numbers of F4/80 positive monocytes and of neutrophils in the spleen sharply rose from d6 to d20 (*p* < 0.001 and *p* < 0.01, respectively) and that in contrast, the number of B cells significantly declined (*p* < 0.01). The number of NK cells in the spleen rose on d14 (*p* < 0.0001) and returned to baseline on d20 (**Figure 3A**). Notably, the changes in immune content of the spleen were overall mirrored in the PB. To illustrate, the number of CD8 T cells in the PB peaked on d10 (*p* < 0.001) and d14 (*p* < 0.01) while the number of CD4 T cells was relatively stable and consequently, the CD8/CD4 ratio in the PB tilted towards CD8 at these time points (*p* < 0.01 for both time points) before decreasing at d20 (*p* < 0.01, **Figure 3B**). Further, as illustrated in **Figure 3B**, the number of neutrophils (*p* < 0.05) in the PB rose from d6 to d14 whereas the number of B cells constantly decreased (*p* < 0.01). Evaluation of the immune content of the BM showed that this organ was also influenced by the tumor. In particular, like the spleen, the BM showed a rise in content of neutrophils and F4/80 positive monocytes, as well as a sharp decrease in content of B cells (**Supplementary Figure 2B**).

#### Comparison of the type and kinetics of the immune response that AB12 cells provoke in the spleen relative to that in TME

To compare the immune response in the spleen relative to that in the TME, we measured in the spleen the expression levels of key genes that we found characterized the immune response changes in the TME across time. As shown in **Figure 3C**, the expression of type I and type II IFNs (and the chemokines CXCL9 and CXCL10 that type II IFN regulates) were at their peak at d6, a finding that echoes well with the enhanced representation of interferon-related pathways in the TME of early-phase tumors. Furthermore, as shown in **Figure 3D**, the expression of perforin-1, granzyme B and to a lesser extent of PD-1 were at their peak on d10, findings that dovetail with the expression patterns found in the TME.

Next, to evaluate the kinetics of the immune response in spleen, we tested *in vitro* whether splenocytes derived from tumor bearing mice respond against AB12 cells in a tumor phase dependent manner. Specifically, we measured the production of IFNg in a co-culture of splenocytes and AB12 cells. As shown in **Figure 3E**, we found that splenocytes of mice with intermediate-phase tumors produced the maximal amounts of IFNg whereas splenocytes of mice with either early- or advanced-phase tumors produced minimal amounts of IFNg (both *p* < 0.0001). Notably, splenocytes of naïve mice did not produce IFNg.

#### Testing the kinetics of the immune response that AB12 cells provoke in the TME and spleen *via* the prism of the response to immune checkpoint inhibitors

Given that anti CTLA-4 has previously been shown to be highly effective against AB12 tumors we selected using this agent to test whether AB12 tumor respond to immunotherapy in a tumor growth phase dependent manner (8, 20).

**Figure 4:**
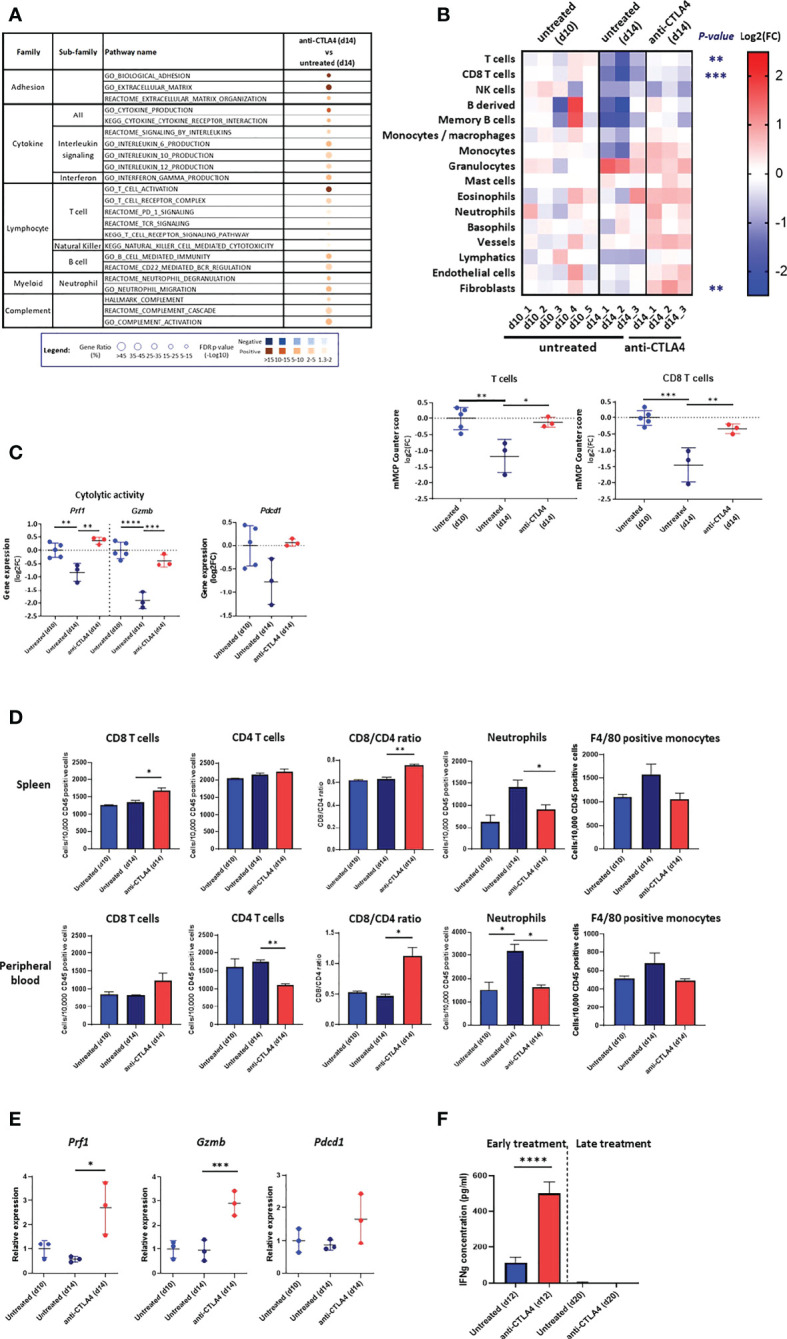
Response to anti CTLA-4 immune checkpoint inhibitor. **(A)** Dysregulated signal pathways in anti CTLA-4 treated AB12 tumors identified by over-representation analysis. Signal pathways representation in AB12 tumors either treated on d6/d10 with anti-CTLA4 (early treatment) or untreated and collected on d14 has been compared. The families and sub-families of the major over-represented signal pathways are shown in the figure. Over-representation of each pathway, based on underexpressed and overexpressed genes, in blue and brown, respectively, is indicated as a circle, whose size is proportional to the gene ratio and the color gradient represents the FDR p-values. **(B)** Differential infiltration of immune and stromal cell populations. In the upper part, the fold-changes (FC) in mMCP-counter scores of each cell population are represented as a heatmap. The collect timepoints and the treatment, and the names of the tumor samples are indicated above and below the heatmap, respectively. The significant *p*-values of the ANOVA test, comparing untreated and anti CTLA-4 treated tumors, are indicated at the left of the heatmap if the FC is higher than 2. In the lower part, the dot plots show the FC for T cell and CD8 T cell populations. The FC are relative to the mean scores obtained in untreated AB12 tumors and collected on d=10. **(C)** Gene expression of cytolytic and activation genes. The dot plots show the FC relative to the mean of d10 tumors of *Prf1* (perforin-1), *Gzmb* (granzyme B) and *Pdcd1* (PD-1) gene expression, based on RNA-Seq data, between anti CTLA-4 treated and untreated tumors. **(D)** Immune content of the spleen and peripheral blood (PB). The numbers, determined by flow cytometry, of CD8 and CD4 T cells, neutrophils and F4/80 positive monocytes out of 10,000 CD45+ cells in the spleen and PB of either untreated d10 and d14 tumor-bearing mice or anti CTLA-4 treated d14 tumor-bearing mice are shown (n>6). The CD8/CD4 ratio at both sites is shown as well. **(E)** Expression of T cells cytolytic and activation genes in the spleen. The relative change in mean expression of the *Prf1*, *Gzmb* and *Pdcd1* genes in the spleens either untreated d10 and d14 tumor-bearing mice or anti CTLA-4 treated d14 tumor-bearing mice are shown (n=3). **(F)** Responsiveness of splenocytes derived from anti CTLA-4 treated mice to AB12 cells *in vitro*. Tumor-bearing mice were either treated early (on d6/d10) or late (on d14/d18) with two injections of anti CTLA-4 and splenocytes were derived from these mice on d12 and d20, respectively, as well as splenocytes from untreated mice. The levels of IFNg protein secreted in the medium were measured by ELISA in splenocytes co-cultured with AB12 cells (n≥4). The *p*-values of the *post hoc* Tukey test and of the unpaired T test are indicated at the top of the dot plots (B to E) and at the top of the histogram **(F)**, respectively. FC: Fold-Change. *p ≤ 0.05; **p ≤ 0.01; ***p ≤ 0.001; ****p ≤ 0.0001.

First, we evaluated how early treatment with anti CTLA-4 (injection on d6 and d10) affected the anti-tumor response by comparing d14 tumors, spleen, and PB from treated mice to d10 and d14 tumors, spleen, and PB from untreated mice. We found that pathways associated with T cells, B cells, neutrophils and NK cells activation, which are all negatively regulated between d10 to d14 in untreated tumors (**Figure 2A**), were all positively regulated in treated d14 tumors relative to untreated d14 tumors (**Figure 4A**, **Supplementary Figure 3A**, **Supplementary Table 3**). Moreover, pathways that are associated with immune mediators and complement were also positively regulated in treated tumors (**Figure 4A**). Notably, treated d14 tumors did not differ from untreated d10 tumors in their pathway activation pattern (**Supplementary Figure 3A**, **Supplementary Table 3**). In line with these findings, we found that the infiltration of T cells and CD8 T cells, determined by mMCP Counter, in treated d14 tumors was similar to their content in untreated d10 tumors but higher than their content in untreated d14 tumors (**Figure 4B**, **Supplementary Figure 3B**, **Supplementary Table 4**). In addition, we found that the expression levels of perforin-1, granzyme B and PD-1 in treated d14 tumors were similar to those observed in untreated d10 tumors, but higher than those observed in untreated d14 tumors (**Figure 4C**).

Concerning the spleen and PB, we found that relative to untreated d14 samples, early treatment with anti CTLA-4 induced an elevation in d14 of the number of CD8 T cells with a shift in CD8/CD4 ratio as well as a reduction in the number of neutrophils and a trend for reduction in numbers of F4/80 positive monocytes (**Figure 4D**). The effect of anti CTLA-4 on the number of NK cells and B cells were less consistent as were its effects on the BM (**Supplementary Figure 3C**). The expression levels of perforin-1, granzyme B and to a lesser extent of PD-1 were elevated in d14 in the spleens of treated vs. untreated mice (**Figure 4E**).

Second, we compared how early treatment and late treatment (injection on d14 and d18) with anti CTLA-4 affected the production of IFNg in the *in vitro* AB12 cells and splenocyte co-culture system described above. We found that early treatment with anti CTLA-4 increased the potential of splenocytes to produce IFNg (*p* < 0.0001) while late treatment with anti CTLA-4 failed to do so (**Figure 4F**).

#### Comparison of the immune response that AB12 cells provoke in the TME to immune states in hMPM

**Figure 5:**
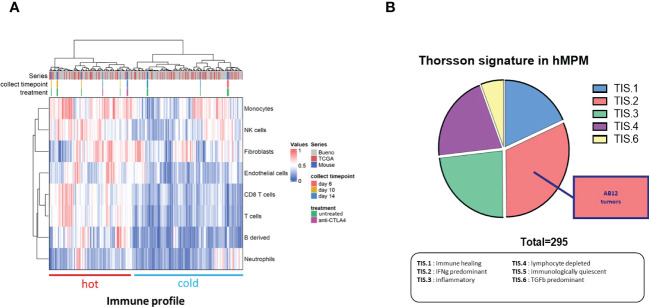
Immune features of AB12 tumors in comparison to hMPM. **(A)** Integration based on immune and stromal cells infiltrations of AB12 tumors in hMPM tumors. Unsupervised clustering of untreated and anti CTLA-4 treated AB12 tumors with 295 hMPM tumor samples was performed based on the infiltrations of immune and stromal cell populations. The series of each tumor sample, the collect timepoint and the treatment of AB12 tumors are indicated by a color code at the top of the heatmap. At the bottom of the heatmap, clusters were divided into tumors with a “hot” and “cold” immune profiles based on immune cells infiltration. **(B)** Thorsson immune classification. Thorsson immune subtypes (TIS) were determined in the 295 hMPM and in AB12 tumor samples using TIS transcriptomic signatures. Tumor samples were classified in one of the TIS based on the highest signature score. The pie chart shows the distribution of the 6 TIS in hMPM tumor samples. AB12 tumors were all classified as TIS2.

To compare the TME of AB12 tumors in each of the tumor growth phases with immune activation states in hMPM, we first used immune content-based unsupervised clustering of human tumors, which allow to separate immune active (“hot”) and immune inactive (“cold”) hMPM as previously described (30) together with murine tumors (**Supplementary Table 4**). As shown in **Figure 5A**, we found that early- and advanced-phase tumors clustered with “cold” hMPM whereas intermediate-phase tumors as well as advanced-phase tumors that were collected from mice that received early treatment with anti CTLA-4 clustered with “hot” hMPM. Next, to determine which hMPM immune subtype the TME of AB12 tumors mimics, we used the Thorsson immune classification of human tumors, which represents the complete immune landscape of human cancer, as a comparative platform (28). Specifically, we determined the tumor immune subtypes (TIS) of all hMPM and all AB12 tumors in our cohort based on the six TIS defined by Thorsson et al. (**Supplementary Table 5**) (28) As shown in **Figure 5B**, TIS.2 (IFNg predominant) was the most common (32%) TIS among hMPM. Furthermore, all AB12 tumors were TIS.2.

### PART 3. Determining the efficacy of standard-of-care anti-hMPM immunotherapy in the model and testing new therapeutic combinations against this benchmark

#### Determining the therapeutic efficacy of standard-of-care anti-MPM immunotherapy in the model

**Figure 6:**
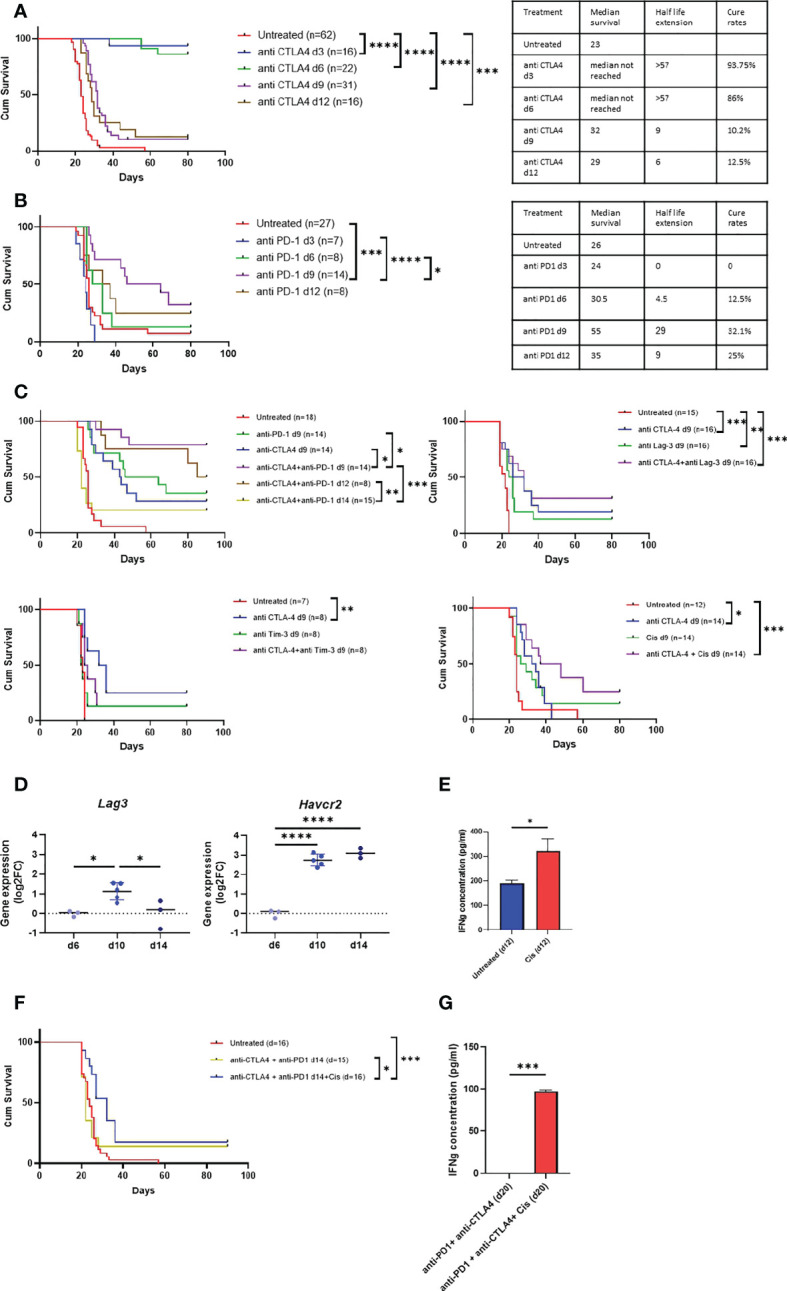
Anti MPM immunotherapy in AB12 tumor model. A-B. Tumor phase dependent efficacy of single agent immunotherapy. The Kaplan-Meier survival curves show the response of AB12 tumors to treatment with either anti CTLA-4 **(A)** or anti PD-1 **(B)** when initiated on d3, d6, d9 or d12. The tables show the median survival, the half-life extension period and the cure rates for each treatment and time point. **(C)** Differential expression *Havcr2* and *Lag3* genes between d6, d10 and d14 AB12 tumors. The dot plots show the fold-changes (FC) of gene expression relative to the mean of d6 tumors of *Havcr2* (TIM-3) and *Lag3* (LAG-3) genes, based on RNA-Seq data. The *p*-values of the *post hoc* Tukey test are indicated at the top of the dot plots. **(D)** Responsiveness of splenocytes to cisplatin treated AB12 cells *in vitro*. AB12 cells were either treated or not with cisplatin. The levels of IFNg protein in the medium were measured by ELISA in tumor cells co-cultured with splenocytes. (n=3). **(E)** Efficacy of combination immunotherapy against AB12 tumors. The Kaplan-Meier survival curves show the response of AB12 tumors to treatment with several combinations of immunotherapy. The responses to anti CTLA-4 + anti PD-1 initiated on d9, or d12 or d14 (upper left), anti CTLA-4 + anti LAG-3 initiated on d9 (upper right), anti CTLA-4 + anti TIM-3 initiated on d9 (lower left), anti CTLA-4 + cisplatin initiated on d9 (lower right) are shown. **(F)** Efficacy of anti CTLA-4 + anti PD-1 + cisplatin against advanced AB12 tumors. The Kaplan-Meier survival curves show the response of AB12 tumors to treatment with either anti CTLA-4 + anti PD-1 or anti CTLA-4 + anti PD-1 + cisplatin initiated on d14. **(G)** Responsiveness of splenocytes derived from anti CTLA-4 + anti PD-1 + cisplatin treated mice to AB12 cells *in vitro.* Splenocytes were derived on d20 from tumor-bearing mice that were treated on d14 with either anti CTLA-4 + PD-1 or anti CTLA-4 + PD-1 + cisplatin. The levels of IFNg protein were measured by ELISA in the medium of tumor cells co-cultured with splenocytes. (n=3). The *p*-values of the unpaired T test are indicated at the top of the histogram (**D, G**). The differences between survival curves were calculated by the log-rank test (**A, B, E**, **F**). FC: Fold-Change; cis: cisplatin. *p ≤ 0.05; **p ≤ 0.01; ***p ≤ 0.001; ****p ≤ 0.0001.

To determine the efficacy of anti CTLA-4 and anti PD-1 in the model either as single or combination therapy, we used survival assay. As shown in **Figures 6A, B**, we found that the efficacy of anti CTLA-4 linearly decreased as the tumor progressed whereas the efficacy of anti PD-1 peaked in d9. Furthermore, we found that anti CTLA-4 showed high cure rates but only in d3 and d6 tumors. In contrast, anti PD-1 showed only moderate cure rates but its maximal potency was in d9 tumors. Based on these observations, we next tested the combination of anti CTLA-4 + anti PD-1 in intermediate phase tumors (d9). We found that the combination was highly effective relative to single agent therapy, raising the cure rates of anti CTLA-4 or anti PD-1 from 28.6%, 35.7% respectively to 78.6% for the combination, and extending the median survival period from 17 and 29 days, respectively, to higher than 57 days for the combination (**Figure 6C** upper left panel, **Supplementary Figure 4A**). Furthermore, we highlighted that anti CTLA-4 + anti PD-1 maintained its efficacy in late-intermediate tumor (d12) but not in advanced tumors (d14).

#### Testing new therapeutic combinations against the standard-of-care efficacy bar

Having determined the efficacy of anti CTLA-4 + anti PD-1 as a preclinical benchmark in the model, we turned to evaluate the relative performance of other combination therapies. First, given that anti CTLA-4 showed very high cure rates in early-phase tumors, we sought to explore whether certain combinations could jumpstart its positive effect also at later time points. Specifically, we tested anti CTLA-4 in combination with either anti LAG-3 or anti TIM-3 or cisplatin. We selected anti LAG-3 and anti TIM-3 because drugs that target them are in advanced clinical development (25) and as shown in **Figure 6D**, their expression levels sharply rise in intermediate-phase tumors in comparison to early-phase tumors. In addition, we selected cisplatin because it is widely used to treat MPM patients (32) and because in our *in vitro* co-culture system, we found that pretreatment of AB12 cells with cisplatin stimulated the production of IFNg by anti-tumor splenocytes (**Figure 6E**).

As shown in **Figure 6C**, in intermediate-phase tumors, combination of anti CTLA-4 with anti LAG-3 or with anti TIM-3 or with cisplatin was ineffective relative to the efficacy of anti CTLA-4 + anti PD-1. However, comparing the efficacy of the combination relative to the efficacy of its separate components, we found that anti CTLA-4 + cisplatin had a marginally significant (*p* = 0.057) synergistic effect relative to either anti CTLA-4 or cisplatin alone. In contrast, anti CTLA-4 + anti LAG-3 showed a minor though insignificant synergism while anti CTLA-4 + anti TIM-3 had no synergism at all (**Figure 6C**).

Based on these observations and on publications showing that in humans anti LAG-3 and cisplatin synergize with anti PD-1 (33, 34), we next tested whether the addition of either of these drugs to the anti CTLA-4 + anti PD-1 combination could improve outcomes in advanced-phase tumors. We found that cisplatin improved the efficacy of standard-of-care in advanced-phase tumors (**Figure 6F**). Moreover, this triple therapy also restored the potential of splenocytes from mice with advanced-phase tumors to produce IFNg *in vitro* upon co-culture with AB12 cells (**Figure 6G**). In contrast, improved efficacy relative to benchmark was not found in the CTLA-4 + anti PD-1 + anti LAG-3 combination (**Supplementary Figure 5A**).

## Discussion

Preclinical models of MM using syngeneic cell lines are used to test and design new anti-hMPM therapeutics (21). However, disparities that exist between MMs and hMPM as well as insufficient characterization of the immunology of MM may result in inaccurate design of preclinical investigations and in premature translation of preclinical findings to the clinics (35). In the current work, comprehensive characterization of the immunobiology of AB12 tumors relative to hMPM as well as calibration of its responsiveness to standard-of-care anti-hMPM immunotherapy generated a systematic three-step approach to improve the utility of the AB12 model as an indicative preclinical tool.

In the first step, we determine the AB12 tumor growth phases showing that based on size and appearance, intermediate- and advanced-phase tumors may be considered as well-established tumors which we suggest are more likely to represent hMPM. Next, based on transcriptomic analysis, we show that in line with their histologic classification (4), AB12 tumors mimic the most common histo-molecular subtype of hMPM: Biphasic (17). In addition, we show that AB12 tumors also represent the most common hMPM immunological subtype: TIS.2 (28). Thus, given that AB12 tumors represents the most prevalent type of hMPM, supports focusing empirical interest on this MM model.

In the second step, we characterize the growth phase-dependent immunobiology of AB12 tumors in the TME and at remote immune sites. With respect to the TME, we show that type I and type II IFN pathways dominate the early-phase of tumor growth. Furthermore, we show that CD8 T cell activation pathways, cytotoxicity genes and immune cell infiltration indices dominate the intermediate-phase of tumor growth while they all decay in advanced tumors. Accordingly, we find that intermediate-phase tumors match “hot” hMPM and that advanced-phase tumors match “cold” hMPM. Based on these findings, and as anticipated by previous works on the role of CD8 T cells in anti-MM responses (8), we can conclude that AB12 cells induce in the TME a bell-shaped, CD8 T cell predominant anti-tumor response that peaks in intermediate-phase tumors. With respect to remote immune organs, we show that AB12 tumors gradually remodel the cellular composition of the spleen, PB and BM, thus indicating that tumor growth has systemic immune effects. In particular, when focusing on the expression of IFNs and cytotoxic genes in the spleen, we demonstrate that the evolution of the anti-tumor response in the spleen and TME has concordant kinetics. This, together with results showing peak production of IFNg by intermediate-phase splenocytes and the potential to enhance IFNg production only by early treatment with anti CTLA-4, substantiates the recognition that immune activation prevails during the early-phase of tumor growth and that profound immune suppression prevails during the advanced-phase of tumor growth.

Together, steps one and two of our approach lay the foundation to evaluate the tumor phase-dependent response to immunotherapy and its efficacy compared to outcomes described in hMPM immunotherapy clinical trials. We approach this in step three, where we show that anti CTLA-4 is highly effective but only in early-phase tumors and that in contrast anti PD-1 is only moderately effective but that it is most potent in intermediate-phase tumors. In addition, we show that standard-of-care combination immunotherapy is superior to single agent therapy in intermediate-phase tumors, and yet, its efficacy declines in advanced-phase tumors. Together, these findings dovetail with clinical trials in hMPM showing that anti CTLA-4 failed to achieve clinical benefit, that anti PD-1 provided marginal clinical benefit with short durations of responses (34), and that CTLA-4 + anti PD-1 provided better results than single agent therapy (1). The overall correspondence between the performance of immunotherapy in the model and in hMPM suggests that the efficacy of standard-of-care in the model can serve as preclinical benchmark that future therapeutics should meet to be considered good candidates for translation to the clinics. Illustrating this principle are our findings on the efficacy of CTLA-4 + anti PD-1 + cisplatin triple therapy relative to benchmark, thereby advocating for its testing in biphasic MPM patients.

From a broader practical perspective, our findings suggest that when testing immunotherapy in the AB12 model, three types of immune effects can be detected depending on time of drug administration. The first immune effect relates to interventions that show efficacy mainly during the *early tumor growth phase*. These interventions should be regarded as ones that are capable of boosting the generation of the anti-tumor response but not necessarily as interventions that can delay the onset of immune suppression or invigorate the anti-tumor response once it has decayed. We predict that such interventions are unlikely to achieve clinical benefit in real-life settings, given that hMPM slowly develop under a strong immunological pressure and because hMPM is often diagnosed in advanced disease stages (13, 14). The second immune effect relates to interventions that show efficacy mainly during the *intermediate phase of tumor growth*, when the anti-tumor response is in its peak. These should be considered as interventions that have the potential to prolong or maintain an existing anti-tumor response. We contemplate that such interventions are most likely to achieve clinical benefit in real-life settings in immune active (“hot”) tumors. The third immune effect relates to interventions that show efficacy also during the *advanced phase of tumor growth*, when the anti-tumor response decays. These should be considered as interventions that have the potential to invigorate an anti-tumor response that has already at least partially been shut down. We propose that such therapies are the most promising with respect to their potential to show clinical benefit in patients with advanced hMPM.

Looking forward from a clinical perspective, our findings that show successful boost in efficacy resulting from the addition of cisplatin to the standard-of care suggests that a promising direction for future research is to explore other potential chemotherapies or targeted therapies to yield even more optimal efficacy. For example, carboplatin offers one potential target given that in the recent checkmate 816 study, it showed greater synergism with anti PD-1 than did cisplatin, in inducing complete response in non-small cell lung cancer patients (33). As another potential target, researchers might explore epigenetic modulators given the synergism that histone deacetylase inhibitors achieved in combination with immunotherapy in non-small cell lung cancer as reported in clinical trials (36). From a preclinical perspective the link between the capacity of AB12 stimulated splenocytes to produce IFNg *in vitro* and the outcomes of *in vivo* survival assays, suggests that a promising direction would be to explore using the AB12 and splenocyte co-culture system as an efficient screen to detect survival enhancing therapeutics prior to *in vivo* testing.

Last but not least, our model focused on biphasic hMPM using the AB12 cell line, however, other murine MM cell lines such as the AB1 (7) and AE17 (22) cells that mimic other histological subtypes of hMPM, also exist. Indeed, using AB1 (6, 24, 37, 38) and AE17 (21, 24) cells as well as AB12 cells (6, 8, 21), past research made significant progress in developing new combination treatments to fight the entire histological spectrum of hMPM subtypes. However, we think that if murine tumor models such as AB1 and AE17 would be assessed and characterized using our approach, the likelihood of successfully translating treatments proposed based on these models to the clinic would increase. To elaborate, promising leads to combining specific chemotherapies and repurposed non-chemotherapeutic drugs with immunotherapy have been made (8, 38). For example, with respect to chemotherapy, using the AB1 cell line, Nowak et al. and Lesterhuis et al. have shown that gemcitabine is not detrimental to antitumor immunity and that it may thus be useful in combination with immunotherapy in general and with anti CTLA-4 in particular (23, 39). In addition, more recently, using both the AB1 cell line and the AE17 cell line Principe et al. have shown that 5-fluorouracil and cisplatin have additive effects when combined with anti CTLA-4 and anti PD-1 (24). With respect to identification of drugs that can be repurposed and combined with immunotherapy, Lesterhuis et al. used network analysis of immunotherapy responsive and irresponsive AB1 tumors to show that hub genes and pathways that are associated with response to immunotherapy can be identified and that drugs that augment or inhibit these hub genes can be effective in combination with immunotherapy. Proof of concept was demonstrated using the nitric oxide generator isosorbide dinitrate to enhance Nitric oxide synthase 2 (NOS2) activity and the small-molecule VX680 to inhibit Aurora Kinase B (AURKB) (38). And yet, these studies were all performed using subcutaneously transplanted MM tumors, presumably to ease follow-up on tumor growth and on response to therapy (6, 8, 20, 22, 24, 38). Furthermore, treatment in these studies was initiated no later than day 12 (in most experiments no later than day 10) and its efficacy was not tested in a tumor growth phase dependent manner. As such, these studies might have tested their interventions in an improper microenvironment or prior to induction of systemic immune suppression by the tumor. Given these considerations, we propose that it may be fruitful for future research to retest these promising leads under orthotopic tumor implantation conditions and using the three-step investigational approach that we applied as a benchmark for calibration of the model and for evaluation of treatment efficacy.

## Conclusions

Our study delineates a systematic approach that improves the capacity of the AB12 model to serve as a screening tool to test and design novel anti biphasic hMPM therapies. We suggest that the efficacy of new therapeutics should be compared to the efficacy of standard-of-care in intermediate- and advanced-phase tumors as these phases more accurately represent hMPM. Therapeutics showing efficacy in advanced-phase tumors are the ones that should be translated to the clinics given their potential to invigorate the anti-tumor immune response even once it has decayed. One such promising combination is the anti CTLA-4 + anti PD-1 + cisplatin triple therapy.

## Data availability statement

The datasets supporting the conclusions of this article are included within the article and its supplementary materials. Raw RNA-seq data are available in Gene Expression Omnibus (GEO) repository under the GSE197542 series number.

## Ethics statement

The animal study was reviewed and approved by Animal Care and Use Committee of the Hebrew University.

## Author contributions

OW, ES and DJ are responsible for the study concept and design. ES, IM, HW performed the experiments. TH, QB and SC developed the bioinformatics tools and the pipeline analysis. ES, OW, SC, CM, and DJ performed the analysis and interpretation of data. OW and DJ were major contributors in writing the manuscript. CT assisted in review and editing of manuscript. OW and DJ are responsible for the study supervision. All authors read and approved the final manuscript.

## Supplementary material

Supplementary Figure 1 Tumor microenvironment in AB12 tumors. **Supplementary Figure 1A**. Comparison of the histologic and molecular subtypes, and the histo-molecular gradients (E.score and S.score) of hMPM tumor samples between the clusters C1 to C3 of the heatmap of . Histograms at the top show the distribution of histologic subtypes and molecular subtypes of hMPM in the TCGA and Bueno series (26, 27). Enrichment of biphasic-like related subtypes is observed in cluster C3 and Chi-square contingency tests highlight significant differential distribution of subtypes between the three clusters (p <0.0001). Box plots at the bottom show the values of the E.score and S.score retrieved from Blum et al. (17). Intermediate values are observed in cluster C3 compared to the two other clusters. The ANOVA tests highlight significant differences between the 3 cluster (p <0.0001) and the p-values of the *post hoc* Tukey test are indicated at the top of the box plots. **Supplementary Figure 1B**. Changes in signal pathways related to alpha and gamma interferon responses and, T cell activation and signaling between d6 to d10 and d14 AB12 tumors. The volcanoplots show the differential mRNA expression between AB12 tumors collected at different timepoints of all the genes included in the pathways: Hallmark_interferon_alpha_response, Hallmark_interferon_gamma_response, GO_T_cell_activation and GO_T_cell_receptor_complex. The collect timepoints are indicated at the top left and right of the volcanoplots. The fold-change (FC) and the adjusted p-values for each gene were retrieved from RNA-Seq data analyzed by DESeq2 package. **Supplementary Figure 1C**. Differential infiltration of immune and stromal cell populations between d6 to d10 and d14 AB12 tumors. The dot plots show the FC relative to the mean of the d6 tumor mMCP counter scores for each of the immune and stromal cell populations between d6, d10 and d14 tumors. Only cell populations showing a significant differential mMCP-Counter scores by ANOVA tests between d6 to d10 or d14 (, left panel) are presented. The p-values of the *post hoc* Tukey test are indicated at the top of the dot plots. **Supplementary Figure 1D**. Gating strategy for AB12 tumor derived cells. Forward and side scatter detector voltage settings were selected based on the position of CD45 + or +CD3 T cell staining. For the detection of T cells, cells of interest were gated for CD45+ and these were split into CD3+ CD8+ and CD3+ CD8- cells. Subpopulation percentages are calculated out of the total number of CD45+ cells. For the detection of CD8+ PD1+ T cells, cells of interest were gated for CD3+ and then for the PD1+CD8+ subpopulation. Subpopulation percentages of CD8+ PD1+ cells were calculated out of the total number of CD3+ cells.

Supplementary Figure 2 Immune response at remote immune sites. **Supplementary Figure 2A**. Gating strategy for spleen, PB and BM derived cells. Forward and side scatter detector voltage settings were selected based on the position of CD45+ cell staining. The gating strategy was anchored on FITC-CD45+ and the percentages of all subpopulations were calculated out of the total number of CD45+ cells. For the detection of T cells, cells of interest were gated for CD45+ and these were split into CD3+ CD8+ and CD3+ CD4+ cells. For the detection of neutrophils, cells of interest were gated for CD45+ and next for CD11b+ and these were split into Ly6G+, Ly6C^high^+ and Ly6C^low^+ cells. For the detection of F4/80 positive monocytes, cells of interest were gated for CD45+ and then were gated for CD11b+ F4/80+ cells. For the detection of NK cells, cells of interest were gated for CD45+ and then were gated for CD3- CD49B+ cells. For the detection of B cells, cells of interest were gated for CD45+ and then were gated for CD19+ cells. **Supplementary Figure 2B**. Changes in the immune content of the bone marrow (BM) of tumor bearing mice between d6, d10, d14 and d20. The numbers, determined by flow cytometry, of CD8 and CD4 T cells, Ly6G+ myeloid cells, NK cells, F4/80 positive monocytes and B cells out of 10,000 CD45+ cells in the BM of tumor bearing mice are shown (n≥6). The CD8/CD4 ratio in the BM is shown as well. Notably in the BM, in contrast to the spleen and PB we determined the numbers of Ly6G+ myeloid cells and not neutrophils since that the used antibody panel permits only making this definition with respect to BM derived cells.

Supplementary Figure 3 Response to anti CTLA-4 immune checkpoint inhibitor. **Supplementary Figure 3A**. Changes in signal pathways related to T cell activation and signaling in anti CTLA-4 treated AB12 tumors. The volcanoplots show the differential mRNA expression between anti-CTLA4 treated and untreated AB12 tumors of all the genes included in the pathways: GO_T_cell_activation and GO_T_cell_receptor_complex. The treatment and the collect timepoints are indicated at the top left and right of the volcanoplots. The fold-change (FC) and the adjusted p-values for each gene were retrieved from RNA-Seq data analysed by DESeq2 package. **Supplementary Figure 3B**. Differential infiltration of fibroblast cell population in anti CTLA-4 treated AB12 tumors. The dot plots show the fold change (FC) of mMCP-counter scores relative to the mean of the untreated d10 tumors for the population of fibroblasts between untreated d10, untreated d14 and treated d14 tumors. Differences in mean fibroblast mMCP-counter scores between treated and untreated tumors were evaluated by ANOVA test, and the p-values of the *post hoc* Tukey test are indicated at the top of the dot plots. *: P ≤ 0.05; **: P ≤ 0.01; ***: P ≤ 0.001; ****: P ≤ 0.0001. **Supplementary Figure 3C**. Immune content of the bone marrow (BM), spleen and peripheral blood (PB) of anti CTLA-4 treated mice. The numbers, determined by flow cytometry, of CD8 and CD4 T cells, Ly6G+ myeloid cells, NK cells and F4/80 positive monocytes out of 10,000 CD45+ cells in the BM of untreated d10 and d14 or anti CTLA-4 treated d14 tumor bearing mice are shown. The CD8/CD4 ratio in the BM is shown as well. The numbers, determined by flow cytometry, NK cells and B cells out of 10,000 CD45+ cells in the spleen and PB of untreated d10 and d14 or anti CTLA-4 treated d14 tumor bearing mice are shown. n>6. Notably in the BM, in contrast to the spleen and PB we determine the numbers of Ly6G+ myeloid cells and not neutrophils since that the antibody panel that we used permits only making this definition with respect to BM derived cells.

Supplementary Figure 4 Summary of the response of tumor bearing mice to immunotherapy. The tables show the median survival, the half-life extension period and the cure rates of tumor bearing mice in response to immunotherapy. The half-life extension period is calculated as the difference in days between the median survival period of untreated mice to the median survival period of mice in the treatment group. Cure rates were determined based on the percentage of mice in each treatment group that survived for more than 80 days. The immunotherapy treatment is listed at the top of each table.

Supplementary Figure 5 Efficacy of anti CTLA-4 + anti PD-1 + LAG-3 against advanced AB12 tumors. The Kaplan-Meir survival curves show the response of AB12 tumors to treatment (two injections per week for a total of two weeks) with either anti CTLA-4 + anti PD-1 or anti CTLA-4 + anti PD-1 + LAG-3 when initiated on d14 (n = 8).

Supplementary Table 1 Flow cytometry data. Table S1.1 List of antibodies for flow cytometry. Table S1.2 Panel of antibodies. Table S1.3 Compensation matrix.

Supplementary Table 2 List of primers.

Supplementary Table 3 Signal pathways enrichment analysis in AB12 tumors. Table S3.1 Differentially expressed genes between untreated mice. Table S3.2 Pathways dysregulated between untreated mice. Table S3.3 Differentially expressed genes between anti CTLA4 treated mice and untreated mice. Table S3.4 Pathways dysregulated between anti CTLA4 treated mice and untreated mice. Pathways are grouped into families and sub-families and the ones over-represented, based on underexpressed and overexpressed genes, are highlighted in blue and brown, respectively.

Supplementary Table 4 Infiltration of immune and stromal cell populations in Tumor microenvironment. Table S4.1 List of biomarkers used in mMCP Counter. Table S4.2 mMCP Counter normalized scores for mouse tumor samples. Table S4.3 List of biomarkers used in MCP Counter. Table S4.4 MCP Counter normalized scores for Human tumor samples.

Supplementary Table 5 Annotations of Human tumor samples.
