# Supplementary material for: Deep dive into the immune response against murine mesothelioma permits design of novel anti-mesothelioma therapeutics
Source: Front Immunol. 2023 Jan 4;13:1026185. doi: 10.3389/fimmu.2022.1026185 (PMC9846605; doi:10.3389/fimmu.2022.1026185)
Supplement: Supplementary Figure 1 — Tumor microenvironment in AB12 tumors. Supplementary Figure 1A . Comparison of the histologic and molecular subtypes, and the histo-molecular gradients (E.score and S.score) of hMPM tumor samples between the clusters C1 to C3 of the heatmap of . Histograms at the top show the distribution of histologic subtypes and molecular subtypes of hMPM in the TCGA and Bueno series (26, 27). Enrichment of biphasic-like related subtypes is observed in cluster C3 and Chi-square contingency tests highlight significant differential distribution of subtypes between the three clusters (p <0.0001). Box plots at the bottom show the values of the E.score and S.score retrieved from Blum et al. (17). Intermediate values are observed in cluster C3 compared to the two other clusters. The ANOVA tests highlight significant differences between the 3 cluster (p <0.0001) and the p-values of the post hoc Tukey test are indicated at the top of the box plots. Supplementary Figure 1B . Changes in signal pathways related to alpha and gamma interferon responses and, T cell activation and signaling between d6 to d10 and d14 AB12 tumors. The volcanoplots show the differential mRNA expression between AB12 tumors collected at different timepoints of all the genes included in the pathways: Hallmark_interferon_alpha_response, Hallmark_interferon_gamma_response, GO_T_cell_activation and GO_T_cell_receptor_complex. The collect timepoints are indicated at the top left and right of the volcanoplots. The fold-change (FC) and the adjusted p-values for each gene were retrieved from RNA-Seq data analyzed by DESeq2 package. Supplementary Figure 1C . Differential infiltration of immune and stromal cell populations between d6 to d10 and d14 AB12 tumors. The dot plots show the FC relative to the mean of the d6 tumor mMCP counter scores for each of the immune and stromal cell populations between d6, d10 and d14 tumors. Only cell populations showing a significant differential mMCP-Counter scores by ANOVA tests between d6 [file DataSheet_1.pdf]

Sup 1

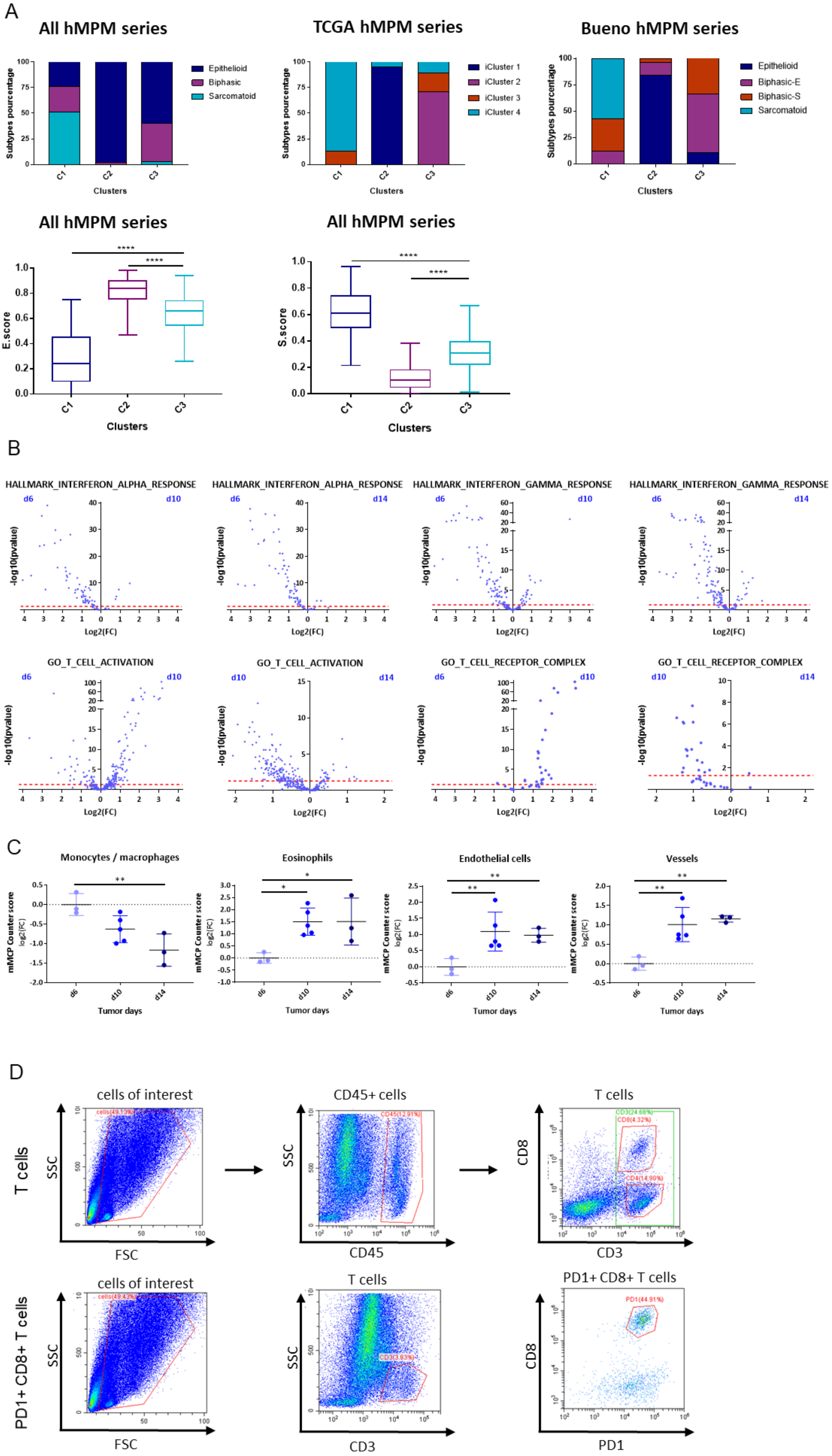
A
All hMPM series
Subtypes pourcentage
Clusters
C1
C2
C3
Epithelioid
Biphasic
Sarcomatoid
TCGA hMPM series
iCluster 1
iCluster 2
iCluster 3
iCluster 4
Bueno hMPM series
Epithelioid
Biphasic-E
Biphasic-S
Sarcomatoid
All hMPM series
E.score
****
All hMPM series
S.score
****
B
HALLMARK_INTERFERON_ALPHA_RESPONSE
HALLMARK_INTERFERON_GAMMA_RESPONSE
GO_T_CELL_ACTIVATION
GO_T_CELL_RECEPTOR_COMPLEX
d6
d10
d14
-log10(pvalue)
Log2(FC)
C
Monocytes / macrophages
Eosinophils
Endothelial cells
Vessels
mMCP Counter score
log2(FC)
Tumor days
d6
d10
d14
**
*
D
T cells
PD1+ CD8+ T cells
cells of interest
CD45+ cells
T cells
PD1+ CD8+ T cells
SSC
FSC
CD45
CD8
CD3
PD1
cells(49.13%)
CD45(12.91%)
CD3(24.68%)
CD8(4.32%)
CD4(14.90%)
cells(49.43%)
CD3(3.83%)
PD1(44.91%)

Sup 2

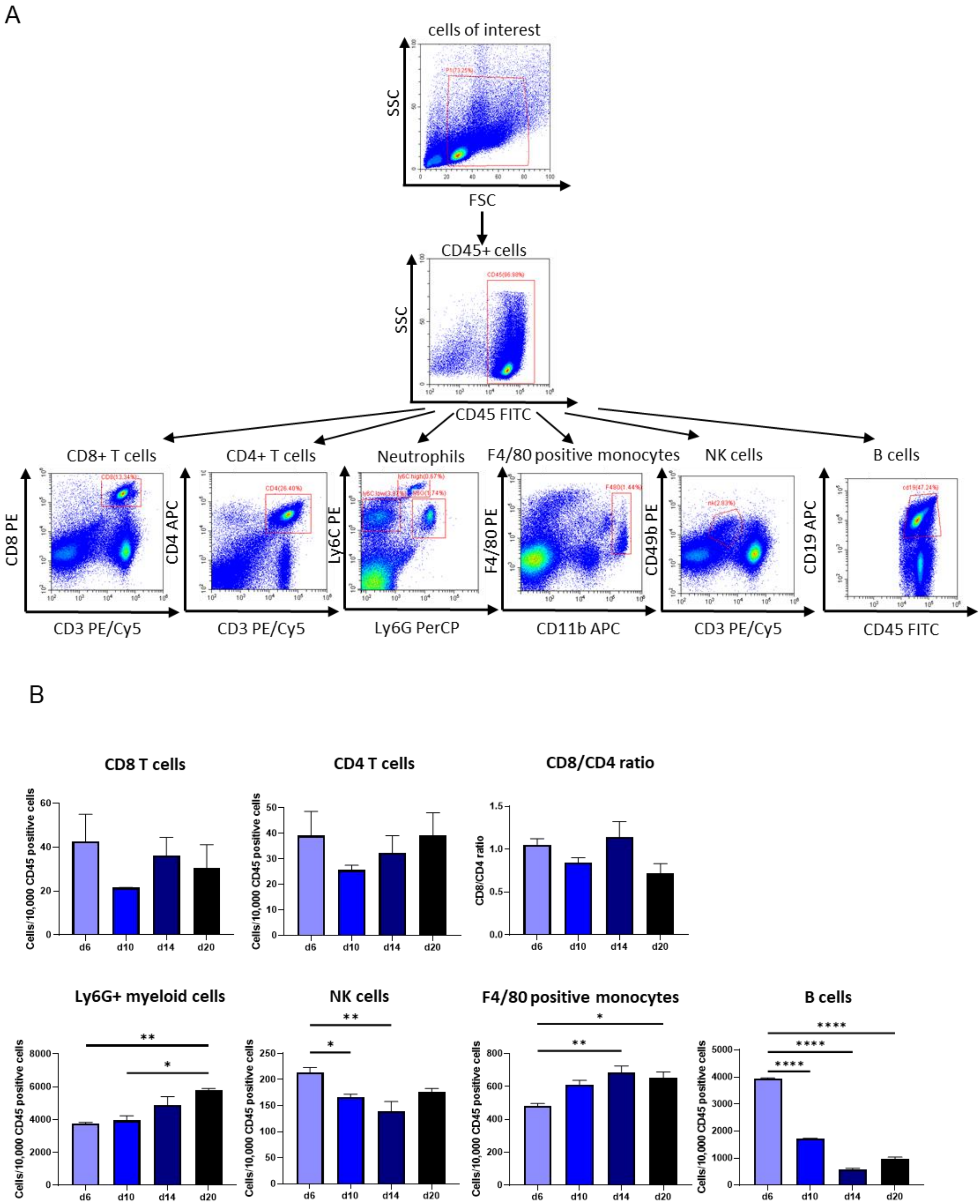
A
cells of interest
SSC
FSC
CD45+ cells
SSC
CD45 FITC
CD8+ T cells
CD8 PE
CD3 PE/Cy5
CD4+ T cells
CD4 APC
CD3 PE/Cy5
Neutrophils
Ly6C PE
Ly6G PerCP
F4/80 positive monocytes
F4/80 PE
CD11b APC
NK cells
CD49b PE
CD3 PE/Cy5
B cells
CD19 APC
CD45 FITC
B
CD8 T cells
CD4 T cells
CD8/CD4 ratio
CD8/CD4 ratio
Ly6G+ myeloid cells
NK cells
F4/80 positive monocytes
B cells
Cells/10,000 CD45 positive cells
d6
d10
d14
d20

Sup 3

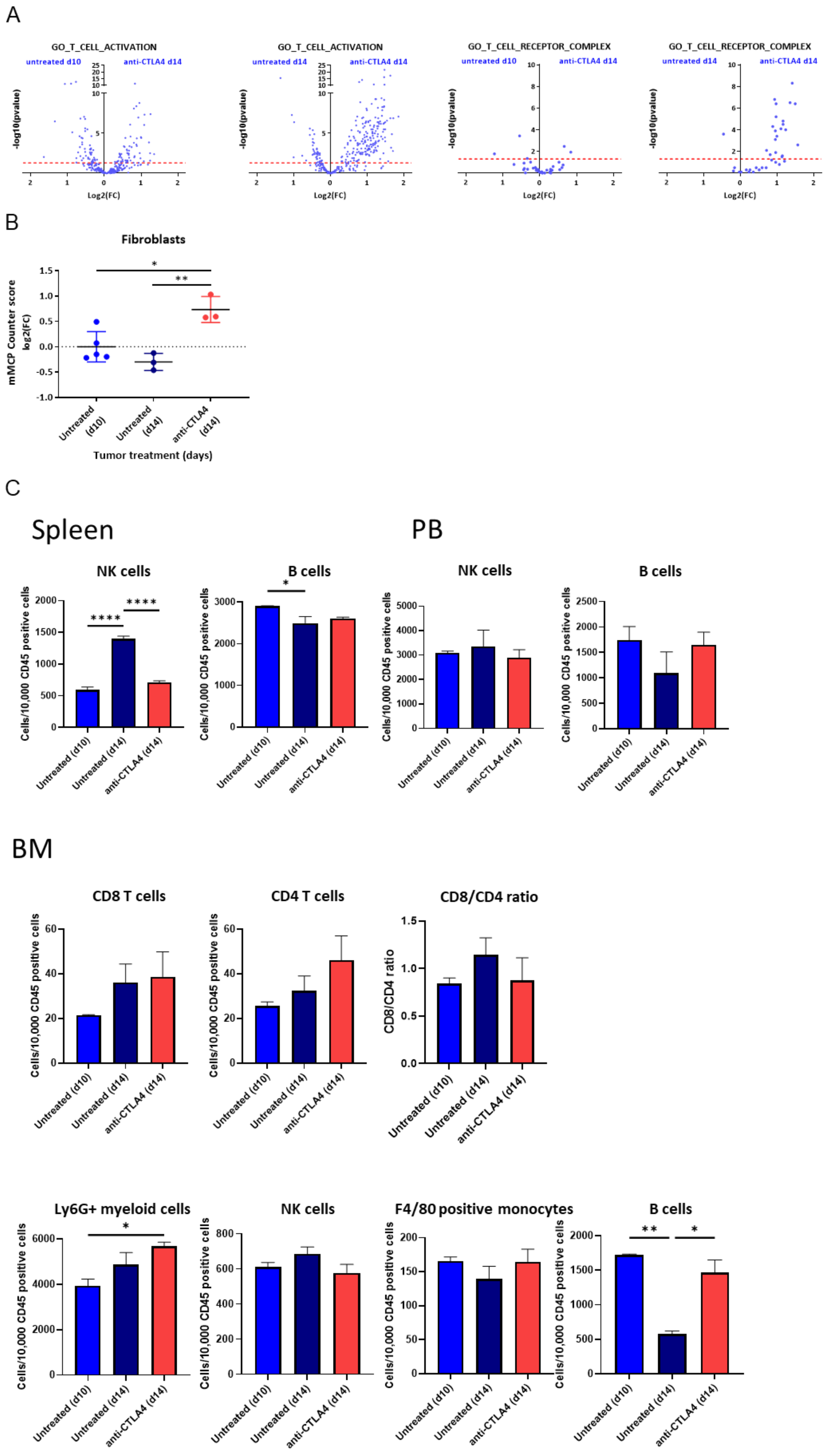

A
GO_T_CELL_ACTIVATION
untreated d10
anti-CTLA4 d14
untreated d14
GO_T_CELL_RECEPTOR_COMPLEX
-log10(pvalue)
Log2(FC)
B
Fibroblasts
mMCP Counter score log2(FC)
Untreated (d10)
Untreated (d14)
anti-CTLA4 (d14)
Tumor treatment (days)
C
Spleen
PB
NK cells
B cells
Cells/10,000 CD45 positive cells
Untreated (d10)
Untreated (d14)
anti-CTLA4 (d14)
BM
CD8 T cells
CD4 T cells
CD8/CD4 ratio
Ly6G+ myeloid cells
NK cells
F4/80 positive monocytes
B cells

Sup 4

anti-CTLA4+ anti-PD1

Treatment	Median survival	Half life extension	Cure rates
Untreated	26		
anti CTLA4 day9	43.5	17.5	28.6%
anti PD1 day 9	55	29	35.8%
anti CTLA4 + anti PD1 day 9	median not reached	>57	78.6%
anti CTLA4 + anti PD1 day 12	80	54	50%
anti CTLA4 + anti PD1 day 14	22	0	20%

anti-CTLA4+ anti-LAG3

Treatment	Median survival	Half life extension	Cure rates
Untreated	22		
anti CTLA4 day 9	29	7	18.75%
anti LAG-3 day 9	25	3	12.5%
anti CTLA4 + anti LAG-3	32	10	31.25%

anti-CTLA4+ anti-TIM3

Treatment	Median survival	Half life extension	Cure rates
Untreated	23		
anti CTLA4 day 9	34	11	25%
anti TIM-3 day 9	22.5	0	12.5%
anti CTLA4+ anti TIM-3	25	2	12.5%

anti-CTLA4+ Cisplatin

Treatment	Median survival	Half life extension	Cure rates
Untreated	24		
anti CTLA4 day 9	33	9	0
Cis day 9	27.5	3.5	14.3%
anti CTLA4+Cis day 9	42.5	18.5	25%

anti-CTLA4+ anti-PD1+Cisplatin

Treatment	Median survival	Half life extension	
Untreated	24		
anti CTLA4 +anti PD1 day 14	22	0	20%
Cis+anti CTLA4 +anti PD1 day 14	32	8	24%

Sup 5

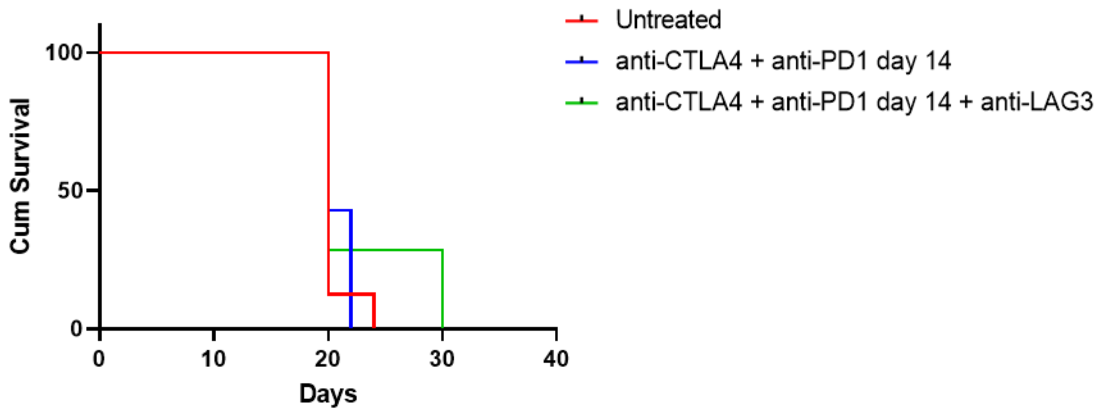
Untreated
anti-CTLA4 + anti-PD1 day 14
anti-CTLA4 + anti-PD1 day 14 + anti-LAG3
Cum Survival
100
50
0
0
10
20
30
40
Days
